# Accumulation of CD4 and CD8 T Cells in Placenta of Malaria Infected Mice Induces the Expression of Hypoxia Inducible Factor-1α (HIF-1α) and Low Birth Weight (LBW) of the Fetus

**Published:** 2019

**Authors:** Zainabur RAHMAH, Teguh WAHJU-SARDJONO, Loeki ENGGAR-FITRI, Adilah ULFIATI, Bougenvil UNGU, Maimun ZULHAIDAH-ARTHAMIN, Eviana NORAHMAWATI

**Affiliations:** 1. Laboratory of Parasitology, Faculty of Medicine and Health Sciences, Universitas Islam Negeri Maulana Malik Ibrahim, Malang, Indonesia; 2. Department of Parasitology, Malaria Research Group, Faculty of Medicine, Universitas Brawijaya, Malang, Indonesia; 3. Master Program in Biomedical Science, Faculty of Medicine, Universitas Brawijaya, Malang, Indonesia; 4. Medicine Study Program, Faculty of Medicine, Universitas Brawijaya, Malang, Indonesia; 5. Department of Clinical Pathology, Dr. Saiful Anwar General Hospital, Faculty of Medicine, Universitas Brawijaya, Malang, Indonesia; 6. Department of Pathology, Dr. Saiful Anwar General Hospital, Faculty of Medicine, Universitas Brawijaya, Malang, Indonesia

**Keywords:** Placental malaria, CD4, CD8, HIF-1α, Low birth weight

## Abstract

**Background:**

Placental malaria involves the sequestration of infected erythrocytes and infiltration of monocytes, helper T cells (CD4), cytotoxic T cells (CD8) as well as T-cell intracellular antigen-1 (TIA-1) in placental intervillous space. These may interferes the nutrient and oxygen transport, causing placental hypoxia and insufficiency that may affect the fetal growth. This study aimed to prove whether the infiltration of lymphocytes in placental malaria mice increases the expression of HIF-1α thus causes fetal Low Birth Weight (LBW).

**Methods:**

Nine pregnant BALB/c mice that infected with *Plasmodium berghei* ANKA strain on day 9 post mating were used as treatment group and 8 non infected pregnant mice were used as control group. The mice were sacrificed on day 18 post mating; then the fetus was weighed individually and the placentas were isolated separately. Expression of CD4, CD8 and HIF-1α were counted by immunohistochemistry using CD4 monoclonal Ab (Santa cruz, sc-59031 CD4) and CD 8 monoclonal Ab (NeoMarker RM-9116-S0) as well as anti-HIF-1α antibody (H1α67) ChIP Grade from Abcam.

**Results:**

There was a higher expression of CD8, CD4 and HIF-1α in infected placenta compare to normal placenta. Analysis using Structural Equation Modeling (SEM) showed expression CD8 and CD4 caused an increase expression of HIF-1α in placenta (t ≥1.96). Expression of HIF-1α caused low fetal weight (t ≥1.96).

**Conclusion:**

In placental malaria, the expression of CD4 and CD8 induce placental hypoxia characterized by increased expression of HIF-1α that causes LBW.

## Introduction

Infection with *Plasmodium falciparum* during pregnancy is associated with accumulation of infected erythrocytes in the placenta, termed as placental malaria that can make extensive adverse effects on the mother and fetus (1). Placental malaria caused by the binding of *P. falciparum* Erythrocytes Membrane Protein-1 (PfEMP-1) on the surface of infected erythrocytes to chondroitin sulfate A (CSA) leading to sequestration of infected erythrocytes in the placental intervilous space, infiltration of inflammatory cells and an increase in pro-inflammatory cytokines (2,3). Histological features of placental malaria are characterized by the presence of malaria parasites and monocytes in intervillous space, the presence of pigment (haemozoin) inside the macrophages, the thickening of the trophoblastic basement membrane (TBM) (4).

Haemozoin (5) and fibrin (6) deposits influence the fetal weight of pregnant mice infected by *P. berghei* however the involvement of lymphocytes cells in placental malaria still unclear although previous study had revealed that there was a role of lymphokine such as IL-17 and IL-10 in placenta malaria (7).

Fetal low birth weight (LBW) is a clinical manifestation which seems to be related with the nutrients and oxygen transport to the fetus (8). In malaria, it may be caused by a high and chronic presentation of parasites in the placental blood stream and placental sequestration of infected erythrocytes associated with cellular immune response (9). All of those may result in mechanical blockage of nutrients and oxygen transport through the placenta (4). This condition may also change the placental function such as inhibiting and disrupting the supply of nutrients and oxygen causing hypoxic effect and impairment of fetal growth (5). The hypoxic placenta will produce hypoxiainducible factor (HIF)-1, a transcription factor that produced as a response to the lack of oxygen in the placenta (10) and may cause the LBW (11). However, the details of these biological processes remain uncertain.

The aim of this study was to prove whether the accumulation of CD4 and CD8 T lymphocytes in the placenta increases expression of HIF-1α and causes fetal LBW.

## Materials and Methods

### Research design and sample

This in vivo experimental laboratory study was conducted using female BALB/c mice weighed 20–30 grams, 13–15 weeks old and healthy. After synchronization the oestrus cycle, the samples then were paired with male mice singly and simultanously mated within one night (7), and then devided into two groups, those were treatment group and control group respectively. Nine mice from treatment group were infected with *P. berghei* intraperitoneally on day 8 ^th^ post mating and 8 mice from control group were not infected. The *P. berghei* ANKA strain used as inoculants in this study were obtained from Laboratory of Parasitology, Faculty of Medicine, Universitas Brawijaya, Malang, Indonesia. The mice then were followed up daily, especially their body weights and pregnancy symptoms, and were sacrificed on day 18 ^th^ post mating. LBW were detected by weighing the entire fetus using analytical Mettler AE 50.

This study was approved by the Ethical Committee of Health Research, the Faculty of Medicine, Universitas Brawijaya (No104/KEPK/7 March 2013) and then conducted at the Laboratory of Parasitology and Laboratory of Biomedics, Faculty of Medicine, Universitas Brawijaya Malang.

### The principles of oestrus synchronization

Oestrus synchronization was done by utilizing the natural phenomenons, namely Lee-Boot effect, Pheromone effect and Whiten effect. Adult rodent females which are housed in groups and isolated from males within certain periods (2–3 weeks) will be suppressed their oestrus cycle and causes them in unoestrus state (Lee-Boot effect). The oestrus cycle will re-start when the un-oestrus females are exposed to male odors by dirty bedding of males (Pheromone effect). The females will be simultaneously in oestrus state about 72 hours after exposing to male odors (Whitten effect) (7).

### Plasmodium berghei ANKA strain inoculation

Inoculation was done by intraperitoneally injection of each mouse as much as 200 μl with parasite concentration 10 ^6^ of *Plasmodium berghei* ANKA strain infected erythrocytes per mL on the day 9 ^th^ post mating, imitating the second period of pregnancy.

### Collection and preparation of placenta and fetus

Isolation of placenta and fetus was done on the 18 ^th^ day post mating. The pregnant mice were scarified under anesthesia with chloroform, and surgery was performed by opening the abdominal wall to take the placenta and fetus. The fetus was weighed individually, and the placentas were fixed with 10% formalde-hyde for immunohistochemistry studies (7).

### Detection of CD4, CD8, HIF-1α Expression

CD4 and CD8 T lymphocytes expression is measured by immunohistochemistry with CD4 monoclonal Ab (Santa cruz, sc-59031 CD4) and CD8 monoclonal antibodies (NeoMarker RM-9116-S0). After all slides were observed, the average number of CD8 cells expression in each group was calculated. The slides were observed under microscopic with a magnification of 1000x using emersion oil. Each slide was observed under 20 fields of view and noted the number of CD4 and CD8 cells expressed. CD4 and CD8 expression was detected as brown color in the cytoplasm of lymphocyte cells that accumulate in the placenta tissue and pale blue to dark blue on the cell nucleus.

Expression of HIF-1α was detected by immunohistochemistry methods using anti-HIF1 alpha antibody (H1alpha 67 Grade from Abcam). Expression of HIF-1α was detected on the intracellular of trophoblast cells in placental tissue. Slides were observed with binocular light microscope under 1000x magnifications on 20 visual fields. The expression of HIF-1α was described by semi-quantitative analysis.

### Data analysis

Data analysis was done using independent-*t* test and Structural Equation Modeling (SEM) method with true tool of Smart Partial Least Square (PLS) software

## Results

### Expression CD8 in placental tissues

**Fig. 1:**
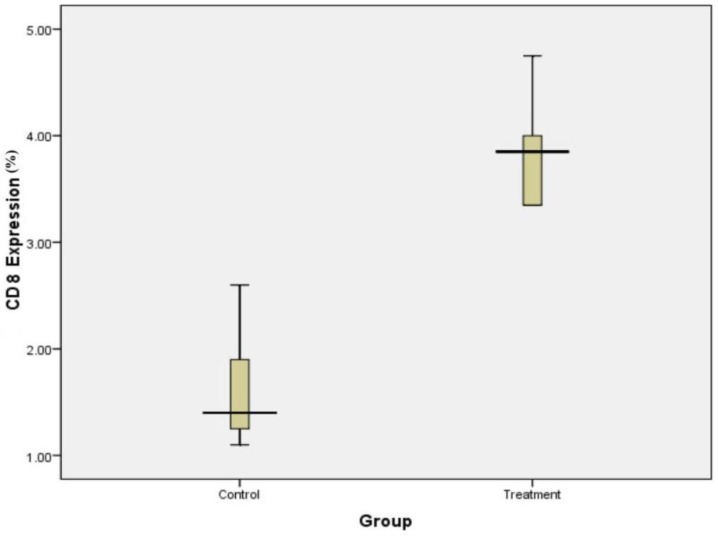
Expression of CD8 in the control and treatment groups. CD8 in the placenta of treatment group was significantly higher than the control group (*P* = 0.001, independent *t* test)

**Fig. 2:**
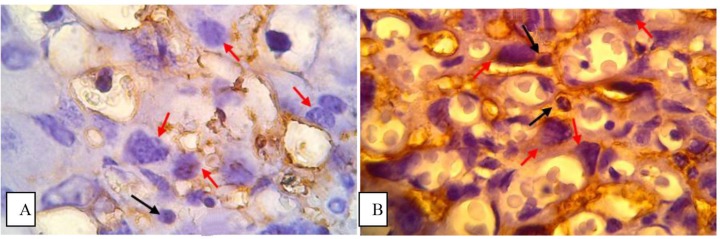
CD8 expression by immunohistochemistry staining. A. The cytoplasm of lymphocytes showed no brown color as CD8 expression in the placenta tissue of control group. B. The cytoplasm of lymphocytes showed a brown color as CD8 expression in the placenta tissue of treatment groups. Black arrows indicate cell lymphocytes and red arrows indicate the trophoblast cells

Expression of CD8 in placental tissues of control and treatment group showed with the following box plot diagram in Fig. 1. The number of CD8 expression was calculated under light microscopy using 1000 x magnification (Fig. 2).

### Expression CD4 in placental tissues

**Fig. 3 :**
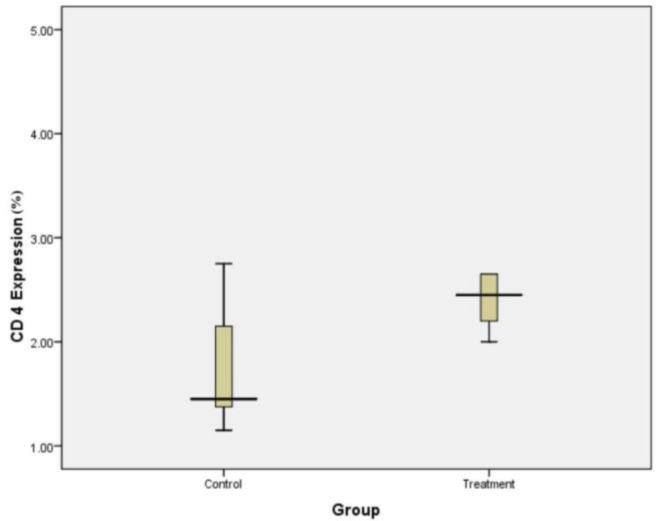
Expression of CD4 in the control and treatment groups. CD4 in the placenta of treatment group was significantly higher than the control group (*P* = 0.009, independent *t* test)

Expression of CD4 in placental tissues of control and treatment group showed with the following box plot diagram in Fig. 3.

**Fig. 4:**
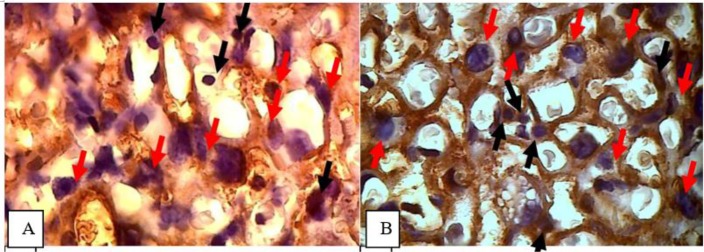
CD4 expression by immunohistochemistry staining. A. The cytoplasm of lymphocytes showed no brown color as CD4 expression in the placenta tissue of control group. B. The cytoplasm of lymphocytes showed a brown color as CD4 expression in the placenta tissue of treatment groups. Black arrows indicate cell lymphocytes and red arrows indicate the trophoblast cells. Magnification 1000x

The number of CD4 expression was calculated using 1000 x magnification light microscopy. As shown in Fig. 4:

### Expression of hypoxia inducible factor-1α (HIF-1α) in placental tissues

**Fig. 5:**
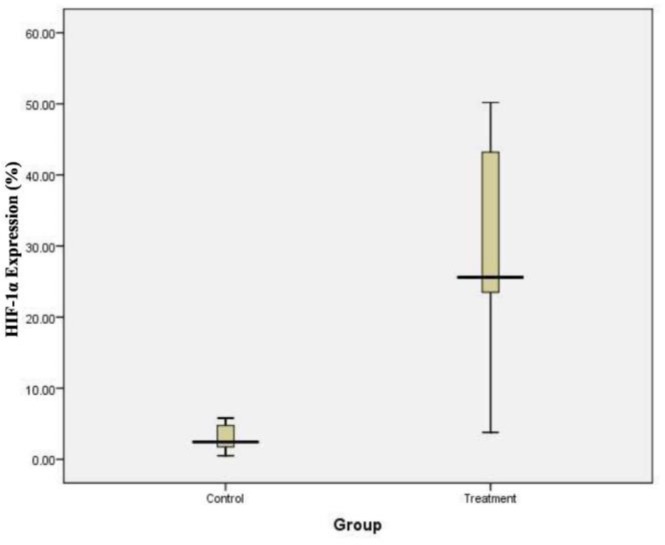
The Expression of hypoxia inducible factor 1α (HIF-1α) in the control and treatment groups. Hypoxia inducible factor-1α (HIF-1α) in the placenta of treatment group was significantly higher than the control group (*P* = 0.01, independent *t* test)

Expression of HIF-1α in placental tissues of control and treatment group showed with the following box plot diagram in Fig. 5.

**Fig. 6:**
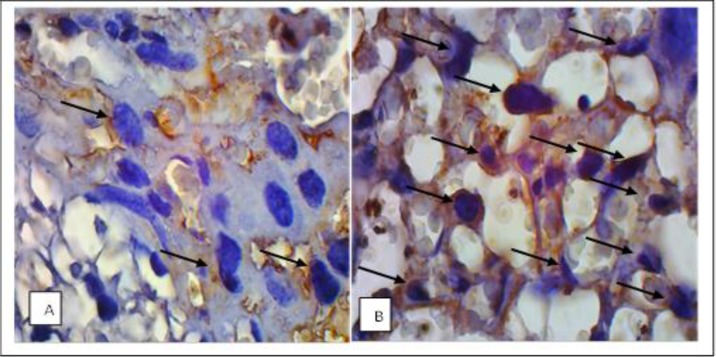
Expression of HIF-1α in placental tissue. A. Control group/non infected pregnant mice. B. The treatment group/infected pregnant mice. Arrows indicate HIF-1α expression in trophoblast cells. Magnification 1000x

The number of HIF-1α in placental tissues of control and treatment group was calculated under light microscopy using 1000 x magnification (Fig. 6).

### Mice fetal weights

**Fig. 7:**
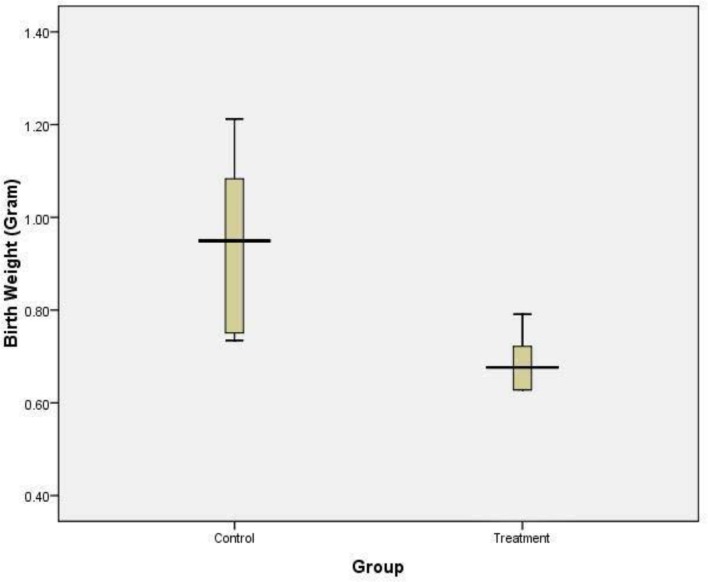
The Fetal weight of the control and treatment groups. The fetal body weight of the treatment group was significantly lower than the control group (*P* = 0.002 independent- *t* test)

**Fig. 8:**
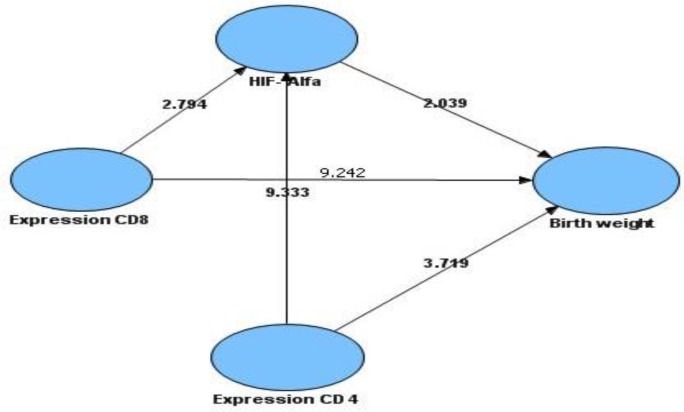
Structural Equation Modeling (SEM) for determining significance on relationship among CD4, CD8, hypoxia inducible factor-1α (HIF-1α) expression and low birth weight with a 95% confidence interval or p = 0.05, which means significant results on the compensation value of t count ≥1,96.

Fetal weights of mice in control group and treatment group were presented on the following box plot diagram in Fig. 7. Data analysis and calculation were done by using the Non-Parametric Structural Equation Modeling and the result can be seen on Fig. 8.

From Fig. 8 showed that CD8 expression had significant relationship to HIF-1α expression in the placenta (t _count_ = 2,794 ≥ t _table_ = 1.96) as well as to birth weight (t _count_ = 9,242 ≥ t _table_ = 1.96) .The expression of CD4 had significant relationship to HIF-1α expression in placenta (t _count_ = 9,333 ≥ t _table_ = 1,96) as well as to birth weight (t _count_ = 3,719 ≥ t _table_ = 1.96). In addition, expression HIF-1α had significant relationship to birth weight (t _count_ = 2,039 ≥ t _table_ = 1,96)

**Fig. 9:**
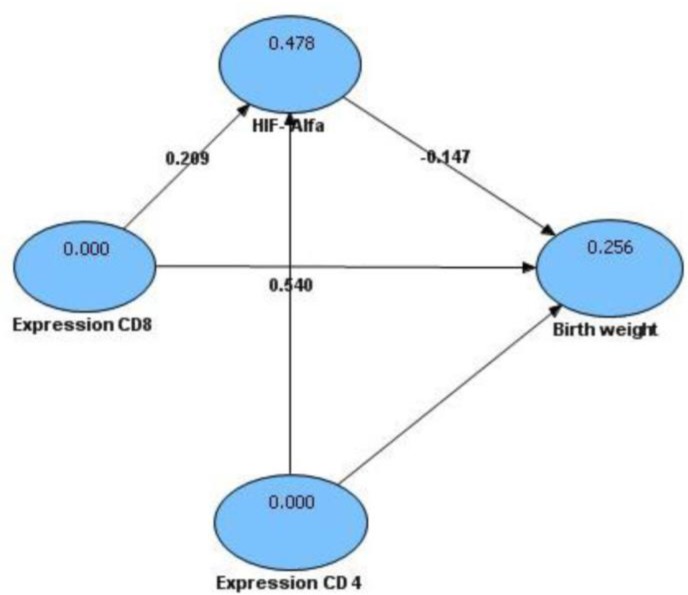
Model hypothesis of the cause-effect relationship among CD4, CD8, Hypoxia Inducible Factor-1α (HIF-1α) and low birth weight, in mice placental malaria illustrated by Value Path Coefficients and R ^2^

A model hypothesis of cause-effect relationship among CD4, CD8, Hypoxia Inducible Factor-1α (HIF-1α) and low birth weight of mice placental malaria is shown in Fig. 9.

From statistical analysis combination of Fig. 8 and 9, it showed that expression CD8 caused an increase expression of HIF-1α in placenta (t _count_ = 2,794 ≥ t _table_ = 1.96; path coefficients = 0.209; R ^2^ = 0.478). The result also showed that the expression of CD4 caused an increase expression of HIF-1α in placenta (t _count_ = 9,333 ≥ t _table_ = 1,96; path coefficients = 0.540; R ^2^ = 0,478). Interestingly CD8 and CD4 did not directly cause low fetal weight, but indirectly through the expression of HIF-1α (t _count_ = 2,039 ≥ t _table_ = 1,96; path coefficients = –0,147; R ^2^ = 0,256).

## Discussion

The first finding of this study showed that the CD4 and CD8 expression was high in the placenta of malaria infected mice. Infected placental parasites cause a significant increase in local immune responses, especially cellular immune response through the accumulation of inflammatory cells in the placenta. Previous study revealed that cellular immune responses in the placenta are dominated by macrophages and monocytes, and there are a number of cytotoxic T cells, especially cells (CD8) and T-cell antigen intracelluler-1 (TIA-1) (12). In *P. falciparum* infected pregnancies, placental sequestration occurs as a result of the accumulation of infected erythrocytes in the intervillous space, infiltration of inflammatory cells and the release of pro-inflammatory mediators (13). This sequestration induces the production of inflammatory mediators such as tumor necrosis factor (TNF-α) (14). In addition besides an increasing level of TNF-α, there is also an increasing of interferon gamma (IFN-γ) level in the placenta and peripheral circulation. Interferon gamma (IFN)-γ is produced by maternal CD4 and CD8 T lymphocytes, natural killer cells, and fetal trophoblast (15).

Accumulation of CD4 and CD8 T lymphocytes in placenta were related with an increasing expression of placenta HIF-1α. Cytoadherence resulted in damage to the wall of the capillary blood vessels and inhibited blood flow to the capillary end as sequestration and rosetting form. The process lead to edema and hypoxia due to capillary leakage and reduced blood flow (16). In hypoxic conditions, HIF-1α is stable because of the blockade of HIF subunit hetero-dimer hydroxylation resulting the transcription of various target genes (17). Hypoxia-inducible factor-1α (HIF-1α) degradation that requires oxygen does not occur due to the low concentration of oxygen in the cells, so that the hydroxylation processes does not occur. Furthermore, the process of HIF-1α poliubiquitination is not going to happen, that makes HIF-1α could not be destroyed by the proteasome (18). Increased HIF-1α transcription in placental malaria indicates the occurrence of a local inflammatory due to the high transcription of HIF-1α occurs in placental syncytium (13). Increased transcription of HIF-1α indicates the occurrence of chronic inflammation or markers of infected erythrocytes in the placenta (19). In this study, high expression of HIF-1α contributed to low fetal weight (20). In the other study, placental malaria causes an increased production of the Th1 subset of CD4 T lymphocytes (21) that cause intrauterine growth restriction and preterm birth (22).

The high density of parasites, chronic infection as well as the contribution of cellular immune improve glucose and oxygen consumption that should be transported to the fetus. In addition, an increase in inflammatory mediators and immune cells that respond to the malaria infection is highly correlated with the occurrence of necrosis and membrane thickening. Syncytiotrophoblast and cytotrophoblast damage allegedly disrupted the supply of nutrients to the fetus (3). Inflammatory cells could lead to functional impairment in placental villi, and disrupted feto-maternal exchange, lead to low birth weight (23, 24). Study in human stated that there was a significant increase in inflammatory cells observed from the placental intervillous obtained from Tanzania country when compared to the placenta from the Spanish state, the non endemic malaria. Accumulation of inflammatory cells, immune cells, and infected erythrocytes in the placenta will impede the flow of blood that carries nutrients to the fetus (placental hypoxia) that will bring harm to the fetus such as low birth weight (12,20).

Hypoxia-inducible factor-1α (HIF-1α) activates the transcription of several vasculogenic factors such as vascular endothelial growth factor (VEGF), VEGF receptor (VEGFR-1 or Flt-1), stromal derived factor-1 (SDF-1), placental growth factor (PlGF) and angiopoitin 1 & 2 and soluble form-like tyrosine kinase-1 (sFlt-1) (25, 26). An increase of HIF-1α, a marker of placental hypoxia correlated with the increase of sFlt-1 (27). Soluble fems-like tyrosine kinase-1 (sFlt-1) will go into the maternal circulation and cause a decrease in VEGF and PlGF. This decrease will result in endothelial dysfunction which then will cause disruption in pregnancy (28) such as fetal growth restriction (FGR) which causes the fetus to experience low birth weight (11).

## Conclusion

Placental sequestration of inflammatory cells induces placental hypoxia indicated by the increase expression of HIF-1α that causes fetal LBW.
